# Effect of Titanium Dioxide Nanotubes on the Shear Bond Strength and Failure Pattern of Metal Orthodontic Brackets Bond to Enamel: An In Vitro Study With Human Teeth

**DOI:** 10.1155/ijod/1315084

**Published:** 2026-06-23

**Authors:** Joviane Jardim Botelho Kemeny Zettl, Érika Soares Bronze-Ulhe, Fabiana Mantovani Gomes França, Francisco Humberto Nociti-Jr, Paulo Noronha Lisboa-Filho, Kamila Rosamilia Kantovitz

**Affiliations:** ^1^ Department of Restorative Dentistry, Faculdade São Leopoldo Mandic (SLMANDIC), Campinas, São Paulo, Brazil, slmandic.edu.br; ^2^ Department of Physics, State University Júlio de Mesquita (UNESP), Bauru, São Paulo, Brazil; ^3^ Division of General Practice, Department of Comprehensive Dentistry, University of Maryland Baltimore School of Dentistry (UMBSOD), Baltimore, Maryland, USA

**Keywords:** adhesive remnant index, glass ionomer cement, nanotechnology, orthodontic brackets, shear bond strength, titanium dioxide

## Abstract

**Background:**

To determine the impact of titanium dioxide nanotubes (nTiO_2_) incorporated into glass ionomer cement (GIC) on its shear bond strength (SBS) and failure pattern of orthodontic brackets bonded to enamel.

**Methods:**

Sixty‐six molars were assigned to three groups (*n* = 22): Transbond XT (TXT) resin; GIC; and GIC + 5% nTiO_2_. SBS was performed using a universal testing machine, and failure patterns were assessed using the adhesive remnant index (ARI). Data were analyzed by generalized linear model and Fisher’s exact test (*α* = 0.05).

**Results:**

TXT showed the highest SBS, followed by GIC and GIC + 5% nTiO_2_ (*p* < 0.05). ARI 0 was the most frequent in all groups, especially in GIC (*p* ≤ 0.05).

**Conclusions:**

Incorporating nTiO_2_ into GIC reduced the SBS and resulted in less material remaining on the enamel after debonding, suggesting a potentially reduced risk of enamel damage.

**Clinical Relevance:**

Orthodontic bracket bonding demands materials that combine reliable adhesion with enamel preservation. Incorporating nTiO_2_ into GIC slightly lowers bracket bond strength but reduces cement remnants on enamel, potentially minimizing enamel damage during debonding. This provides clinicians with a well‐supported alternative for orthodontic bonding that balances reliable adhesion with enhanced enamel preservation.

## 1. Introduction

Orthodontic treatment is essential for improving the dental alignment and overall oral health [1]. However, challenges such as white spot lesions [2] and bracket bond failure [3] can compromise outcomes and patient satisfaction [4]. White spot lesions, caused by biofilm‐induced enamel demineralization, affect up to 97% of patients within the first month, while bracket bond failure has a reported prevalence of up to 37%, causing treatment delays and reduced success [4]. While composite resins are widely used for bracket bonding due to their ease of handling and strong adhesive properties [5], they have key limitations such as no anticaries properties and poor mechanical performance in moist or contaminated environments [6]. In this context, resin‐modified glass ionomer cements (RMGICs) could be an alternative to composite resins to attach orthodontic brackets to enamel, especially in cases of high‐risk caries populations, combining anticaries benefits and mechanical properties less affected by intraoral contaminants [7, 8].

Previous studies by our group have demonstrated that titanium dioxide nanotubes (nTiO_2_) significantly affected the mechanical and biological properties of a conventional GIC. Overall, the findings of these studies demonstrated that the addition of nTiO_2_ to the GIC matrix improved several key properties, including microhardness [9], compressive strength [10], opacity [10], solubility [11], and radiopacity [12] without adversely affecting surface roughness [9] or adhesion to dental substrates [10]. Intriguingly, nTiO_2_ into the GIC matrix also modulated the expression of cariogenic genes in dental biofilm models [13, 14]. A synergistic antimicrobial effect of GIC and TiO_2_ nanostructures was later confirmed by meta‐analysis [15]. While titanium dioxide nanoparticles have been studied in orthodontic adhesives like Transbond XT (TXT) [16], their incorporation into GIC for bonding brackets to enamel has not been previously investigated. In the current study, it was hypothesized that a GIC incorporated with nTiO_2_, featuring improved mechanical properties, would serve as a reliable approach to bind orthodontic brackets to the enamel. In vitro assays were performed to determine the in vitro performance of a GIC incorporated with nTiO_2_ by assessing its shear bond strength (SBS) and failure pattern of metal orthodontic brackets bonded to human enamel in comparison with a composite resin and GIC alone.

## 2. Material and Methods

### 2.1. Sample Size and Materials

**Figure 1:**
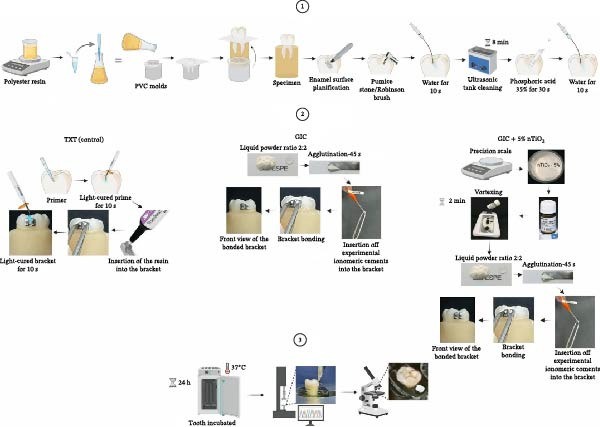
Experimental design: (1) specimens preparation—a precise scale weighted the GIC powder and the nTiO_2_, each tooth root was embedded in acrylic resin within PVC molds, the surface was flattened and the enamel was etched; (2) bonding procedures—bracket was positioned and fixed on the tooth with different materials; (3) specimens storage (24 h), shear bond strength test and failure pattern analyses.

This in vitro experimental study was approved by the Ethics Committee (CAAE: 69268723.4.0000.5374). Sample size (*n* = 22/group) was calculated for 80% power at *α* = 0.05 using G∗Power 3.1. Sixty‐six molars were randomly assigned to three groups: (1) TXT, (control), (2) Ketac Molar EasyMix (GIC), and (3) GIC + 5% nTiO_2_. SBS (MPa) and adhesive failure pattern (adhesive remnant index [ARI]) were assessed (Figure 1). Table 1 describes the materials, compositions, manufacturers, and batch numbers. Nanotubes (~20 nm length and ~10 nm diameter) were synthesized via the alkaline method and characterized as previously described [17]. The 5% nTiO_2_ concentration was based on previous studies [9–14] using the same particles and GIC, showing improved physicomechanical properties without affecting adhesion, surface roughness, or antibacterial activity. nTiO_2_ was weighed (0.625 g), manually mixed into GIC powder, and homogenized in a vortex for 2 min [10]. SEM and TEM confirmed uniform dispersion without agglomeration [12]. All analyses were performed by a single calibrated operator.

**Table 1:** Materials, composition, manufacturer, and batch numbers of the materials used in the present study.

Material	Composition	Manufacturer	Batch number
Transbond XT	Treated silane quartz 70%–80%, bisphenol A diglyceryl ether dimethacrylate (Bis‐GMA) 10%–20%, bisphenol dimethacrylate A bis (2‐hydroxyethyl ether) 5%–10%, treated silane silica <2%, diphenyliodonium hexafluorophosphate <1%. Triphenylantimony <1%.	3M/ESPE, St. Paul, MN, USA	2228300750
Transbond Light Cure Adhesive Primer	Treated silane quartz, bisphenol A diglyceryl ether dimethacrylate (BISGMA), dimethacrylate bis (2‐ hydroxyethyl ether bisphenol A, treated silane silica, diphenyliodonium hexafluorophosphate.	3M Unitek Orthodontic Products South Peck Road, CA, USA	NF18498
Ketac Molar EasyMix	Powder: glass powder composed of aluminum fluorosilicate, calcium and lanthanum, polyacrylic acid, tartaric acid, ascorbic acid, benzoic acid and pigments (shade A3). Liquid: water, acrylic acid copolymer and maleic, tartaric acid, benzoic acid.	3M Deutschland, Neuss, Germany	9144742/9073600
Metal bracket	Orthometric advanced edgewise brackets, 0.022″ slot, stainless steel, upper central incisor	Orthometric, Marília, SP, Brazil	152758001/157185001
Ultra‐Etch 35% phosphoric acid	35% phosphoric acid	Ultradent, South Jordan, UT, USA	BLL2H

### 2.2. Teeth Selection

Sixty‐six erupted caries‐free human third molars extracted for orthodontic reasons were selected. The teeth were cleaned, stored in 0.5% chloramine T at freezing temperature, and examined under 20× magnification. Inclusion criteria were sound, freshly extracted teeth without prior chemical treatment; teeth with defects, wear, cracks, fractures, caries, or white spot lesions were excluded (ISO 11405:2018) [18] (Figure 1).

### 2.3. Specimen Preparation

The tooth root was embedded in acrylic resin (PVC tubes), with its longitudinal axis aligned to the tube. Enamel surfaces were flattened using 400‐, 600‐, and 1200‐grit papers (Arotec S.A., Ind. and Com., São Paulo, Brazil) under water cooling and then polished with 1.0 μm diamond paste (Buehler Metadi II, Buehler, Lake Bluff, IL, USA). Buccal surfaces were cleaned with a Robinson brush, pumice, and water for 10 s, rinsed, and dried for 10 s. Brushes were replaced every five teeth, and specimens were ultrasonically cleaned for 8 min (Figure 1).

TXT and GIC, with or without nTiO_2_, were applied to stainless‐steel brackets (0.022″ edgewise slot prescription, straight base, and welded wings; Orthometric, Marília, SP, Brazil). The brackets were positioned at the center of the crown with tweezers (Ice, São Paulo, SP, Brazil), excess material was removed using an explorer #5, and all bonding was performed by a single trained operator.

For the TXT group, enamel was conditioned with 35% phosphoric acid (30 s), rinsed (30 s), and air‐dried (2 s). Transbond Light Cure Adhesive was applied and light‐cured for 10 s. Resin composite was applied directly on the bracket base, and both were light‐cured for 10 s from vertical and lateral directions, at a 2 mm distance, using the standard mode of the Valo Grand light‐curing unit (1000 mW/cm^2^).

For GIC groups, material agglutination was performed using a metallic spatula and a waterproof paper pad [19]. The powder and liquid were accurately weighed to ensure a consistent 1:1 powder‐to‐liquid ratio across all experimental groups, as recommended by the manufacturer. The mixture was immediately applied to the bracket base using a Centrix syringe. Specimens were coated with a thin petroleum jelly (Rioquímica, São José do Rio Preto, SP, Brazil, batch #1702146) and stored for 24 h at 37°C.

### 2.4. SBS Test

SBS was performed using a universal testing machine (EMIC, DL200 Ind. e Com. LTDA, Campinas, SP, Brazil) at 1 mm/min, following ISO 29022 [20] (Figure 1). The chisel‐shaped tip applied force perpendicularly to the tooth‐bracket interface in the occlusocervical direction (Figure 1). SBS (MPa) was calculated as SBS = *F*/*A*, where *F* is the maximum force required to debond (N) and *A* is the bracket bonding area (mm^2^). Values were converted from kgf/cm^2^ to MPa.

### 2.5. Failure Pattern Analyses (ARI‐Qualitative Analysis)

After SBS testing, failures were evaluated under a stereomicroscope at 20x (SQF‐F, Tecnival, São Paulo, SP, Brazil) using the ARI [21]: 0 = no material on enamel; 1 = ≤50% on enamel; 2 = >50% on enamel; 3 = all material on enamel with bracket imprint. A blinded, calibrated examiner performed all analyses twice, 1 week apart, with Spearman’s correlation showing a 91% agreement.

### 2.6. Statistical Analysis

Data were analyzed using Shapiro–Wilk and Levene‘s tests (*p* < 0.05). Generalized linear models analyzed SBS data, while Fisher’s exact test assessed failure patterns (in %) (*α* ≤ 0.05) using a software (R Core Team [2022]. R: A language and environment for statistical computing. R Foundation for Statistical Computing, Vienna, Austria).

## 3. Results

### 3.1. SBS Test Analysis

Intergroup data analysis demonstrated that SBS was significantly affected by the type of material used as well as by the incorporation of nTiO_2_ into the GIC matrix. The resin composite group (TXT) featured the highest SBS values (31.83 MPa) followed by GIC alone (17.62 MPa) and GIC + 5% nTiO_2_ (10.04 MPa) (*p* < 0.05). Therefore, the presence of nTiO_2_ significantly reduced the SBS values in comparison with those of the GIC group alone. Table 2 illustrates the SBS findings.

**Table 2:** Mean (standard deviation) and median (minimum; maximum) of the shear bond strength from enamel to orthodontic metal brackets (MPa) based on experimental groups (*n* = 22).

Experimental groups	Mean (standard deviation) (in MPa)	Median (minimum; maximum) (in MPa)
TXT (control)	31.83 (10.67)^a^	32.09 (10.50; 49.22)
GIC	17.62 (3.67)^b^	18.44 (11.17; 23.11)
GIC + 5% nTiO_2_	10.04 (3.96)^c^	9.67 (2.75; 18.05)

*Note:* Different letters indicate a significant difference among experimental groups by generalized linear models (*p* < 0.0001).

### 3.2. Failure Pattern Analysis

**Figure 2:**
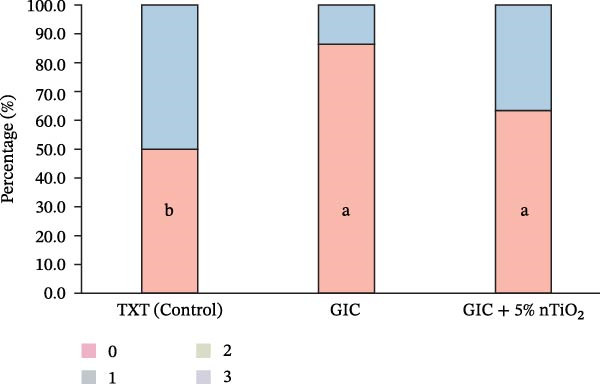
Distribution of failure patterns according to the adhesive remnant index (ARI) in the experimental groups. Different letters indicate statistically significant differences among groups, according to the generalized linear models and Fisher’s exact tests (*p* < 0.05). ARI were defined as: 0 = no material on enamel; 1 = ≤50% on enamel; 2 = >50% on enamel; 3 = all material on enamel with bracket imprint.

In the current study, only scores 0 and 1 were found. Intergroup statistical comparisons showed that the resin composite group (TXT) displayed the lowest percentage of score 0 (50%) in comparison with GIC (86.4%) and GIC + nTiO_2_ (63.6%), whereas score 1 was found to be higher in the TXT group (50%) followed by GIC + nTiO_2_ (36.4%) and GIC (13.6%) (*p* < 0.05). Together, these findings demonstrate that the TXT group displays a higher adhesion to the enamel than GIC and that nTiO_2_ did not significantly impact the failure pattern of the GIC group. Figure 2 illustrates the ARI test results, while Table 3 shows the representative images of enamel surfaces after SBS testing for each experimental group. Images illustrate the predominant failure patterns observed after debonding.

**Table 3:** Representative images of enamel surfaces after SBS testing for each experimental group.

TXT (control)	GIC	GIC + 5% nTiO_2_
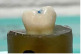 ARI = 0	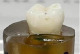 ARI = 0	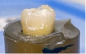 ARI = 0
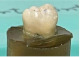 ARI = 1		

*Note:* Images illustrate the predominant failure patterns observed after debonding. 0 = no material on enamel; 1 = ≤50% on enamel; 2 = >50% on enamel; 3 = all material on enamel with bracket imprint.

## 4. Discussion

Orthodontic bonding is influenced by several factors, including dental substrate condition, bracket design, orthodontic forces, and adhesive materials. Effective bonding relies on proper substrate preparation, stress distribution, and adhesive application to ensure bond strength and durability throughout the orthodontic treatment [6]. This in vitro investigation evaluated the SBS and ARI of metal brackets bonded with GIC‐containing 5% nTiO_2_ on sound enamel in comparison with GIC alone and a resin composite (TXT). While nTiO_2_ did not significantly improve SBS, it slightly decreased the frequency of ARI score 0 compared to that of GIC alone, suggesting that nTiO_2_ has the potential to affect GIC’s failure patterns to enamel. Regarding the SBS analysis, it was observed that TXT featured the highest levels, followed by GIC and GIC + 5% nTiO_2_ (*p* < 0.0001). All materials displayed SBS values within the clinically acceptable range (5.9–7.8 MPa), supporting their potential clinical use.

Understanding the dental bonding mechanism is crucial to determine a given material’s performance and prevent adhesive failures. Resin‐based composites bond via micromechanical interlocking and hybridization through resin monomer infiltration into the demineralized enamel [22], whereas GICs bond both chemically and micromechanically through ionic interactions between calcium ions in hydroxyapatite and carboxyl groups in polyacrylic acid [23]. Due to their hydrophilic nature, GICs enhance wettability, facilitating bonding [23]. However, the bonding process is slower and results in an ion‐exchange‐resistant layer [21, 23]. Previous reports have shown that GIC without acid conditioning (10% polyacrylic acid) results in lower bond strength compared to that of resin‐based adhesives [24].

It has been shown that while nTiO_2_ does not chemically interact with the GIC matrix, it enhances certain physical properties like microhardness [9] and fluoride release [9] while reducing wear and solubility [10]. In the current study, GIC‐containing nTiO_2_ featured reduced SBS compared to GIC alone and TXT. These results are similar to earlier investigations showing that nanomaterials like chitosan and silver significantly reduced GICs’ SBS values [24, 25] and may potentially be explained by an inverse relationship between fluoride release and the material’s mechanical strength [26]. While nTiO_2_ has been reported to increase GIC adhesion to the dentin [10], it seems to have no significant effect on enamel bonding, most likely because enamel’s density and low‐porosity structure (96% hydroxyapatite, 10–200 nm crystals) limit GIC penetration [27, 28]. On the other hand, dentin’s higher porosity (with features such as 60 nm intertubular space and 25 nm peritubular dentin) allows for cement penetration and physical bonding, aided by dentinal fluids [29].

In the current study, standardized stainless‐steel orthodontic brackets with large bases were used to ensure uniform bonding and greater resistance to orthodontic forces [30]. ARI analysis revealed only scores 0 (no material on the enamel) and 1 (≤50% material remaining), with GIC alone featuring the highest percentage of scores 0 (86.4%), followed by GIC + 5% nTiO_2_ (63.6%) and TXT (50%) (*p* < 0.05). Clinically, the lower adhesive remaining on enamel in the GIC groups suggests a reduced risk of damage during debonding, minimizing microfractures or demineralization, and facilitating post‐removal cleanup. The addition of 5% nTiO_2_ did not affect this protective effect, maintaining a balance between adequate bond strength and enamel preservation. In contrast, the higher adhesion of TXL may increase the risk of enamel damage, highlighting the clinical advantage of GIC for preserving enamel integrity.

While in vitro studies cannot fully simulate the oral environment, they provide useful insights for clinical research. Further in vitro and in vivo studies, including thermocycling, should be considered to further explore the potential benefits of nTiO_2_ added to GICs to support its use for orthodontic treatment, especially in high‐risk caries populations.

## 5. Conclusion

It can be concluded that nTiO_2_ decreased the SBS of metal orthodontic brackets bonded to the enamel using a GIC, whereas nTiO_2_ slightly altered the frequency of complete material removal after bracket debonding. Additional in vitro and in vivo studies must be designed to further support the clinical use of modified GIC to support bracket installation during orthodontic treatment.

## Funding

No funding was received for this manuscript.

## Conflicts of Interest

The authors declare no conflicts of interest.

## Data Availability

The data that support the findings of this study are available from the corresponding author upon reasonable request.
